# Exposed Implant after Immediate Breast Reconstruction – Presentation and Analysis of a Clinical Management Protocol

**DOI:** 10.1055/s-0041-1735939

**Published:** 2021-10-20

**Authors:** Rafael Amin Menezes Hassan, Cícero de Andrade Urban, Maíra Teixeira Dória, Cleverton Cesar Spautz, Iris Rabinovich, Karina Furlan Anselmi, Eduardo Schunemann Jr, Flávia Kuroda, Bernardo Passos Sobreiro, Rubens Silveira de Lima

**Affiliations:** 1 Department of Post-graduation, Universidade Positivo, Curitiba, PR, Brazil; 2 Breast Unit, Hospital Nossa Senhora das Graças, Curitiba, PR, Brazil; 3 Department of Obstetrics and Gynecology, Universidade Federal do Paraná, Curitiba, Paraná, PR, Brazil

**Keywords:** breast neoplasm, breast reconstruction, exposed implant, infection, neoplasia de mama, reconstrução da mama, exposição de implante, infecção

## Abstract

**Objective** Infection and exposure of the implant are some of the most common and concerning complications after implant-based breast reconstruction. Currently, there is no consensus on the management of these complications. The aim of the present study was to review our cases and to present a clinical protocol.

**Methods** We conducted a retrospective review of consecutive patients submitted to implant-based breast reconstruction between 2014 and 2016. All patients were managed according to a specific and structured protocol.

**Results** Implant exposure occurred in 33 out of 277 (11.9%) implant-based reconstructions. Among these, two patients had history of radiotherapy and had their implant removed; Delayed reconstruction with a myocutaneous flap was performed in both cases. Signs of severe local infection were observed in 12 patients, and another 5 presented with extensive tissue necrosis, and they were all submitted to implant removal; of them, 8 underwent reconstruction with a tissue expander, and 2, with a myocutaneous flap. The remaining 14 patients had no signs of severe infection, previous irradiation or extensive tissue necrosis, and were submitted to primary suture as an attempt to salvage the implant. Of these, 8 cases (57.1%) managed to keep the original implant.

**Conclusion** Our clinical protocol is based on three key points: history of radiotherapy, severe infection, and extensive tissue necrosis. It is a practical and potentially-reproducible method of managing one of the most common complications of implant-based breast reconstruction.

## Resumo

**Objectivo** Infecção e exposição da prótese são algumas das complicações mais comuns e preocupantes após reconstrução da mama com implantes. Atualmente, ainda não há consenso quanto ao manejo destas complicações. O objetivo deste estudo foi o de revisar os casos da nossa instituição e apresentar um protocolo clínico.

**Métodos** Realizou-se uma revisão retrospectiva de todos os casos consecutivos submetidos a reconstrução mamária imediata com prótese entre 2014 e 2016. Todos os casos foram conduzidos de acordo com um protocolo específico e estruturado.

**Resultados** A exposição do implante ocorreu em 33 de 227 reconstruções (11,9%). Dentre estas, duas pacientes tinham histórico de radioterapia, e foram submetidas a remoção da prótese e posterior reconstrução com retalho miocutâneo. Sinais de infecção local grave foram observados em 12 pacientes, e, em 5, necrose extensa de tecido, e todas foram submetidas a remoção dos implantes; destas, 8 foram reconstruídas com expansor, e 2, com retalho miocutâneo. As 14 pacientes remanecentes não haviam sido submetidas previamente à radioterapia, não tinham sinais de infecção, nem necrose extensa; portanto, foram submetidas a sutura primária em uma tentativa de salvar a prótese. Dessas, 8 pacientes (57,1%) conseguiram manter os implantes originais.

**Conclusão** Nosso protocolo clínico é baseado em três pontos principais: histórico de radioterapia, infecção grave, e necrose extensa de tecido. Ele constitui um método prático e potencialmente reprodutível de manejo de uma das complicações mais comuns da reconstrução mamária com implantes.

## Introduction

The rate of postmastectomy breast reconstruction (PMBR) has increased worldwide. 1 2 3 4 5 In the United States, there was an increase of 35% between 2000 and 2017, with more than 100 thousand procedures performed in 2017. 6 In Brazil's public health system, the rate of PMBRs increased from 15% in 2008 to 29% in 2014. 3 Breast reconstruction is associated with cosmetic and psychosocial benefits, and improvements in quality of life. 7 8 9 10 Among the different types of breast reconstruction, implant-based surgery is the most common option. 1 2 11 Several studies 12 13 14 15 have already demonstrated that this type of reconstruction is not associated with a negative impact on the oncologic results of breast cancer treatment, or with an increased risk of developing postoperative complications when compared with mastectomy alone.

Of all possible complications, implant infection and exposure remain major concerns, as they can lead to implant loss and bad cosmetic results. 7 16 17 18 The rate of implant infection varies between 1% and 35.4%, and exposure occurs in 0.25% to 8.3% of all implant-based breast reconstructions. 19 20 21 22 23 Several factors are associated with implant infection and exposure: chemotherapy, radiotherapy, tumor size, obesity, older age, axillary dissection, smoking, and the comorbidities of the patient. 16 19 24 25

Traditionally, implant infection is treated with antibiotic therapy, removal of the implant, and delayed reconstruction. 23 26 27 28 29 30 More recently, cases of implant salvage have been reported. 11 23 26 29 30 31 32 However, there is no consensus on the definition of implant salvage and on the clinical management of this situation. Device salvage might be defined as maintaining the implant itself, or the implant pocket, or even as salvage of the reconstructive result. 11 The attempt of saving the implant may include only systemic antibiotics, or antibiotics associated with a surgical procedure (wound drainage, pocket lavage, capsulotomy, and implant exchange). 11 26 29 31 32 To salvage exposed implants, authors report capsular flap coverage, 33 34 device exchange with primary suture, 26 31 or device exchange with muscular flap. 26 31

In face of the lack of structured and clear information concerning the management of patients with implant exposure with or without infection after breast reconstruction, in the present manuscript, we review our cases and provide a clinical roadmap for the management of these patients.

## Methods

We conducted a retrospective review of consecutive patients submitted to implant-based breast reconstruction between January 1st, 2014, and June 30st, 2016. Mastectomies were performed by one of seven surgeons of Breast Unit of Hospital Nossa Senhora das Graças, in the city of Curitiba, Southern Brazil. The same surgeon performed all breast reconstructions and managed the complications. Preoperative antibiotic prophylaxis with 2 g of Cefazolin was administrated in every case. Clindamycin was used for patients allergic to β-lactams. We used implants from two manufacturers: Allergan plc (Dublin, Ireland), and Mentor Worldwide LLC (Irvine, CA, United States). The study was approved by the local Ethics Committee (under protocol no. 178.554).

The following demographic data were analyzed for each patient: age, presence of comorbidities, body mass index (BMI), smoking, previous breast surgeries, chemotherapy, radiotherapy, and axillary dissection. Regarding the surgical technique, the following data were evaluated: type of mastectomy (nipple-sparing or skin-sparing mastectomy), use of autologous tissue, timing of reconstruction (immediate or delayed), type of protheses (definitive or temporary), and breast weight. The type of reconstruction was defined individually for each patient by the surgical team, considering oncological staging, the patients' desire, biophysical characteristics, type of surgery, risk factors, and adjuvant treatment. Patients submitted to breast reconstruction with a different team of surgeons, those submitted to cosmetic surgeries, those with follow-up shorter than 3 months, and patients initially submitted to reconstruction with a myocutaneous flap were excluded.

**Fig. 1:**
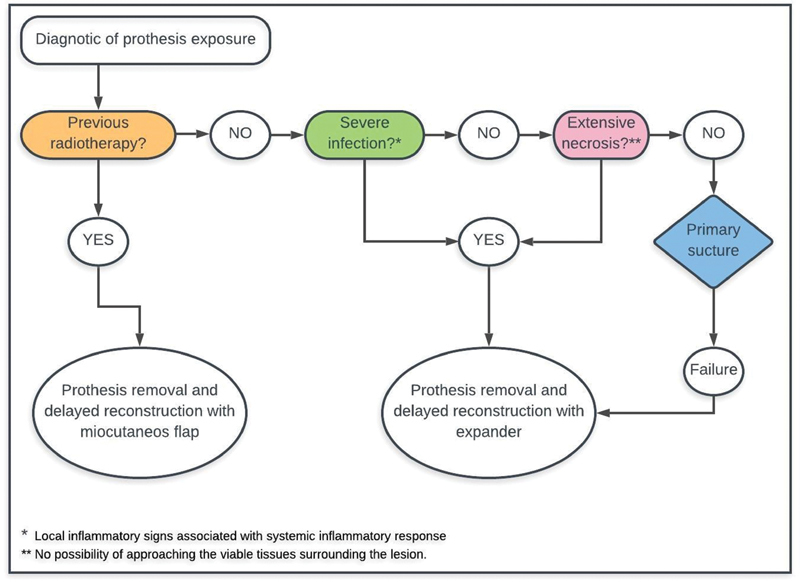
Management protocol for exposed implants after immediate breast reconstruction.

The patients were treated according the same management protocol ( Fig. 1 ). Briefly, after identifying implant exposure, three factors are evaluated: previous irradiation, presence of infection, and presence of necrosis. For patients previously submitted to radiotherapy, implant removal and myocutaneous reconstruction is indicated. Primary suture or local flap advancement are indicated for those that have not received irradiation, have no signs of severe infection, and have minor necrosis. Patients presenting severe infection and/or extensive necrosis are submitted to implant removal and delayed reconstruction with a tissue expander after at least 3 months. If there is another failure in this second procedure, reconstruction with a myocutaneous flap is indicated.

Severe implant infection was defined as local inflammatory signs (erythema, edema, cellulitis, local warmth), with or without purulent discharge, associated with systemic inflammatory response (fever, leukocytosis, or hypotension). Necrosis of the flap was defined as absence of vitality of the overlying tissue, causing loss of function. Extensive necrosis is defined when there is no possibility of approaching the viable tissues surrounding the lesion.

### Statistical Analysis

Descriptive data was presented as frequencies and percentages. The Pearson Chi-squared test was used to compare the rates of complications of each risk factor, and Fisher exact test was used when necessary. The Student *t* -test was used for the continuous variables, and the Mann-Whitney U test, for the ordinal variables. Values of *p* < 0.05 were considered statistically significant. The software used was the Epi Info (Centers for Disease Control and Prevention, Atlanta, GA, United States), version 7.

## Results

A total of 277 mastectomies with implant-based reconstruction were performed in 232 patients in the period analyzed. Of the 45 contralateral mastectomies performed, 32 (71.1%) were prophylactic (11 with proven breast cancer related-mutations, and the others with family history of breast cancer or presence of atypical lesions), and 13 (28.9%) were oncologic (synchronic tumors). The mean follow-up was of 19.2 months.

Table 1 shows the clinical and epidemiological characteristics of the study cohort. The mean age was of 50.2 years (range: 23 to 84 years), and 83% of the patients were younger than 65 years of age at the day of the surgery. Most patients did not have any comorbidities (79.4%), and had BMIs of up to 25 kg/m ^2^ (64.3%). Of all patients, 12.3% had history of smoking, and 27.4% received neoadjuvant chemotherapy. In total, 56 patients had 1 or more complications (20.2%); of these, 36 needed hospitalization. The most common complication was implant exposure ( *n* = 33; 11.9%), followed by small tissue necrosis ( *n* = 27; 9.4%), severe infection ( *n* = 18; 6.5%) and extensive necrosis ( *n* = 7; 2.5%). The mean time between surgery and prosthesis exposure was of 7 weeks.

**Table 1:** Clinical and epidemiological characteristics of the study cohort

Characteristic	Number of patients (%)
*Age in years (standard deviation)*	50.2 (10.56)
*Menopausal status*	
Premenopausal	167 (60.3%)
Postmenopausal	110 (39.7%)
*Comorbidities*	
None	220 (79.4%)
Cardiovascular disease	45 (16.2%)
Diabetes	16 (5.8%)
*History of smoking*	
Yes	34 (12.3%)
No	243 (87.7%)
*Body mass index*	
≤ 25 kg/m ^2^	178 (64.3%)
25–30 kg/m ^2^	66 (23.8%)
≥ 30 kg/m ^2^	33 (11.9%)
*Type of mastectomy*	
Skin-sparing mastectomy	80 (28.9%)
Nipple-sparing mastectomy	197 (71.1%)
*Previous breast surgery*	
Yes	55 (19.9%)
No	222 (80.1%)
*Radiotherapy*	
Prior to surgery	12 (4.3%)
After surgery	42 (15.2%)
No	224 (80.9%)
*Neoadjuvant chemotherapy*	
Yes	76 (27.4%)
No	201 (72.6%)
*Mean weight of the breast (grams)*	408.06
*Complications*	
No	221 (79.8%)
Yes	56 (20.2%)
Prothesis exposure	33 (11.9%)
Small-tissue necrosis	27 (9.4%)
Severe infection	18 (6.5%)
Extensive necrosis	7 (2.5%)

Table 2 shows the comparison between patients with and without implant exposure. No associations were found regarding age, menopausal status, comorbidities, history of smoking, previous breast surgery, radiotherapy, type of surgery, type of prothesis, mean weight of the breast, and manufacturer of the prothesis. A higher proportion of BMIs between 25 kg/m ^2^ and 30 kg/m ^2^ was found among patients with exposure than among those without it (30.3% ( *n* = 10 out of 33) versus 22.9% ( *n* = 56 out of 244) respectively), as well as a higher proportion of BMIs above 30 kg/m ^2^ (21.2% versus 10.7% respectively), but these findings were not statistically significant (odds ratio [OR] = 1.68; 95% confidence interval [95%CI]: 0.72–3.90; *p* = 0.17; and OR = 2.35; 95%CI: 0.90–6.18; *p* = 0.06 respectively). Among patients with implant exposure there was also a higher proportion of axillary dissection (27.3% ( *n* = 9 out of 33) versus 18.9% ( *n* = 46 out of 244) among patients without exposure), although this difference was not statistically significant.

**Table 2:** Univariate analysis between the two groups

Characteristic	Prothesis exposure ( *n* = 33) (%)	No exposure ( *n* = 244) (%)	Odds ratio (95% confidence interval)	Fisher exact
*Menopausal status*				
Premenopausal	20 (12.0%)	147 (88%)	1.01 (0.48–2.12)	NS*
Postmenopausal	13 (11.8%)	97 (88.2%)		
Comorbidities**				
None	24 (11.0%)	196 (89.0%)		
Diabetes	2 (12.5%)	14 (87.5%)	1.16 (0.25–5.45)	NS
Cardiovascular disease	8 (18.0%)	37 (82.0%)	1.76 (0.74–4.23)	NS
*History of smoking*				
Yes	5 (14.7%)	29 (85.3%)	1.32 (0.47–3.70)	NS
No	28 (11.5%)	215 (88.5%)		
*Body mass index*				
≤ 25 kg/m ^2^	16 (9.0%)	162 (91.0%)		
25–30 kg/m ^2^	10 (15.1%)	56 (84.9%)	1.80 (0.77–4.21)	NS
≥ 30 kg/m ^2^	7 (21.2%)	26 (78.8%)	2.72 (1.02–7.26)	NS
*Previous breast surgery*				
No	29 (13.1%)	193 (86.9%)	0.52 (0.17–1.55)	NS
Yes	4 (7.3%)	51 (92.7%)		
*Radiotherapy*				
No	28 (12.6%)	195 (87.4%)		
Prior to surgery	1 (8.3%)	11 (91.7%)	0.63 (0.07–5.09)	NS
After surgery	4 (9.5%)	38 (90.5%)	0.73 (0.24–2.21)	NS
*Neoadjuvant chemotherapy*				
Yes	10 (13.1%)	66 (86.9%)	1.17 (0.53–2.59)	NS
No	23 (11.4%)	178 (88.6%)		
*Mean weight of the breast (grams)*	453.7	397.4	*p* = 0.197***	
*Type of mastectomy*				
Skin-sparing mastectomy	11 (13.8%)	69 (86.2%)	1.26 (0.58–2.75)	NS
Nipple-sparing mastectomy	22 (11.1%)	175 (88.9%)		
*Type of prothesis*				
Silicone	20 (13.4%)	129 (86.6%)		
Temporary expander	2 (11.8%)	15 (88.2%)	1.16 (0.24–5.47)	NS
Definitive expander	11 (10.1%)	98 (89.9%)	1.38 (0.63–3.01)	NS
*Prothesis Manufacturer*				
Allergan plc	19 (12.3%)	135 (87.7%)	1.05 (0.50–2.20)	NS
Mentor Worldwide LLC	14 (11.8%)	105 (88.2%)		
*Axillary dissection*				
Yes	9 (16.4%)	46 (83.6%)	1.68 (0.73–3.88)	NS
No	23 (10.4%)	198 (89.6%)		

Abbreviation: NS, not significant.

Notes: *The Fisher exact test was considered not significant when *p* > 0.05. **The percentage is over 100% because some patients had 2 or more comorbidities. ***The Student *t* -test was used for the comparative analysis of the means.

**Fig. 2:**
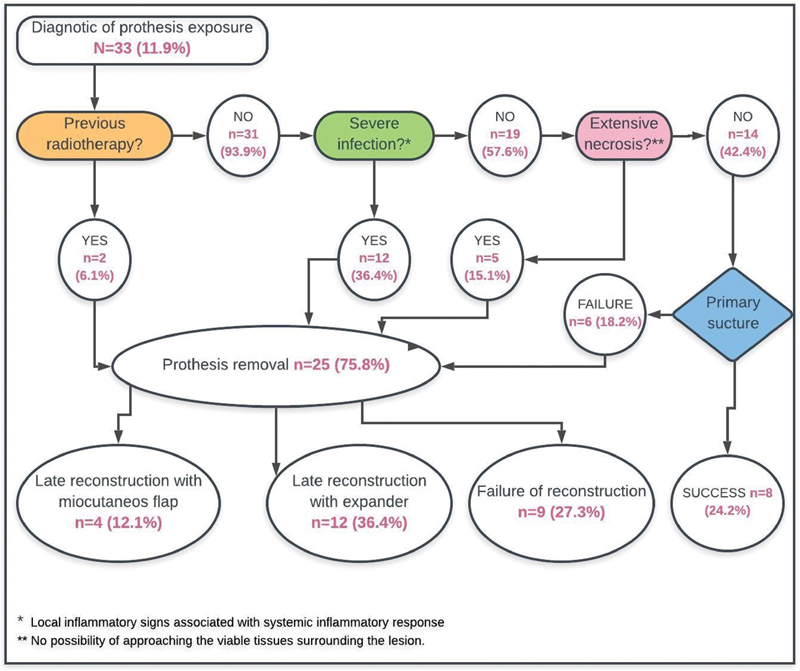
Clinical management of the study cohort according to the protocol.

**Fig. 3:**
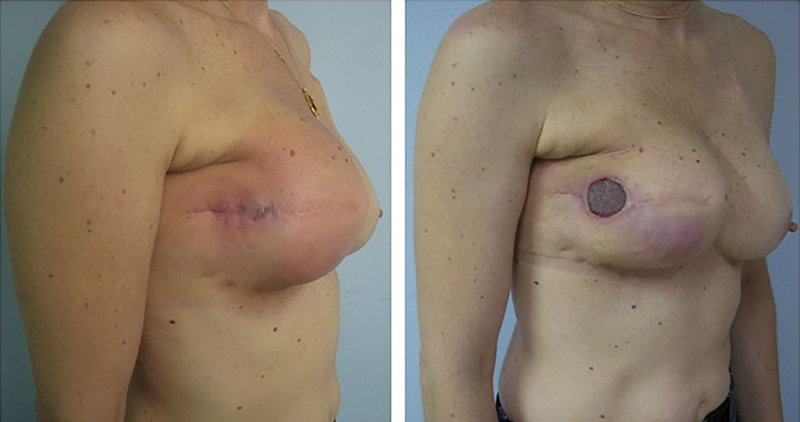
Temporal evolution of extrusion in an irradiated patient.

Figure 2 illustrates the protocol for the management of all patients with implant exposure. The first question is if the patient had been previously submitted to radiotherapy. Of the 33 cases of exposed prosthesis, 2 (6.1%) patients had history of radiotherapy and had their devices removed ( Fig. 3 ); delayed reconstruction with a myocutaneous flap was performed in both cases. The remaining 31 patients had not received radiotherapy, and were evaluated for signs of severe infection. The answer was affirmative in 12 cases, and they were submitted to implant removal. At the end of the follow-up, 4 of these patients had undergone reconstruction with a tissue expander, and 1 (8.3%), with a myocutaneous flap. The remaining 7 patients (58.3%) either chose not to proceed with the delayed reconstruction ( *n* = 6), or did not have success with the second attempt ( *n* = 1).

**Fig. 4:**
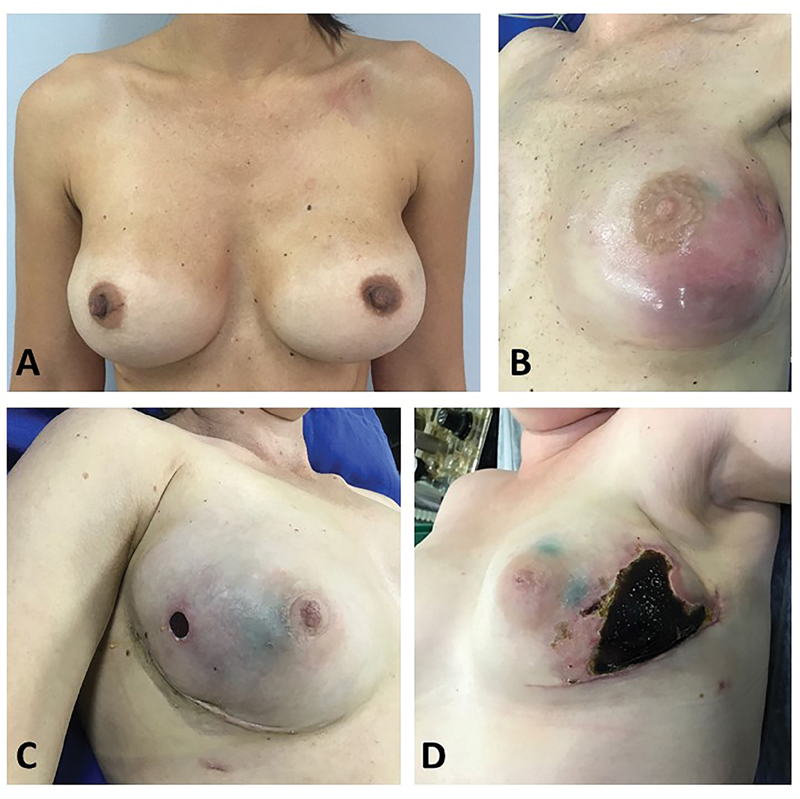
Postoperative photographs after immediate reconstruction. ( **A** ) Good result after immediate reconstruction. ( **B** ) Local severe infection. ( **C** ) Minor exposure of the prothesis. ( **D** ) Extensive necrosis.

In total, 19 patients had no signs of severe infection or previous irradiation; of these, 5 (26.3%) presented with extensive tissue necrosis ( Fig. 4 ). All of them were initially submitted to removal of the device. After the removal, 4 (66.7%) patients underwent reconstruction with a tissue expander, and 1 (16.7%), with a myocutaneous flap. One of these patients died due to the oncologic disease. The remaining 14 (42.4%) patients had no signs of severe infection, previous irradiation, or extensive tissue necrosis, and were submitted to primary suture as an attempt to salvage the implant ( Fig. 4 ). Of these, the original implant was kept in 8 cases (57.1%). Of the remaining 6 patients, 3 (50%) changed the implant for a tissue expander; 2 (33.3%) choose not to reconstruct the breast; and 1 (16.7%) was submitted to reconstructiona with myocutenoues flap due to the bad quality of the skin.

At the end of the follow-up, reconstruction was successful in 24 (72.7%) out of 33 patients with prosthesis exposure. Considering all 277 patients, our success rate was of 96.7% ( *n* = 268).

## Discussion

Immediate breast reconstruction after mastectomy has become a widely-accepted surgical option, and it can yield good cosmetic results, improving the quality of life of the patients. 9 Surgical complications, such as implant exposure and/or infection, may result in the removal of the device, additional surgical procedures, bad cosmetic results, and psychological suffering for the patients. 7 16 17 18 29 Therefore, precise management of these complications is imperative. In this manuscript, we presented a clinical protocol for exposed implants with and without associated infection. We also reviewed all cases of immediate implant-based reconstruction to evaluate the application of this protocol.

Our complication rate of 20.2% is similar to that reported in the literature. 16 18 25 35 Of all complications, prosthesis exposure was the most common, and was present in 11.9% of our patients (33 out of 277 implant-based reconstructions). This percentage is slightly higher than that described by other authors, which ranges from 0.25% to 8.3%. 19 20 23 Our high percentage of immediate reconstructions with definitive implants may be an explanation for this discrepancy. We have not found any statistically significant factors associated with prothesis exposure; this might be due to the small number of cases, as other authors have already demonstrated that smoking, radiotherapy, tumor size, obesity, older age, axillary dissection, chemotherapy, and patient comorbidities are associated with complications of reconstructions. 16 19 24 25 Our rate of implant loss of 9.0% (25 of 277 implant-based reconstructions) is comparable to those published in the literature, which vary from 0.9% to 13%. 16 17 18

Our clinical protocol to manage the complications of implant-based reconstruction is based on clinical parameters and on the experience of a single surgeon. The first parameter is the history of radiotherapy. If the patient has been irradiated before, the exposed prosthesis must be removed. Bennett et al. 31 evaluated 68 patients (with a total of 71 implant-based breast reconstructions) who developed infection or skin necrosis/exposure over a 20-year period. The patients were treated in one of three ways: explantation with or without delayed reconstruction; explantation with or without immediate autologous reconstruction; or implant salvage. Of the 20 patients submitted to the attempt to salvage the implant, 65% underwent radiotherapy prior to their complication. The implant was successfully kept in 4 (30.8%) out of 13 patients with a history of radiotherapy, and in 5 (71.4%) out of 7 with no history of radiotherapy. The authors 31 concluded that patients previously submitted to radiotherapy have a higher rate of success when the size of the implant is reduced, or when new tissue, such as a flap, is introduced.

The second parameter to be analyzed is if the patient with the prosthesis exposed presents signs of severe infection. In that case, the implant is removed, and the patient receives systemic antibiotics. A delayed reconstruction is proposed after at least three months. Most authors who investigated the possibility of salvaging infected implants excluded patients with severe infection. 11 31 32 Spear and Seruya 26 reported their 15-year experience with the management of infected or exposed breast protheses after reconstructive or cosmestic surgery. A total of 69 patients with 87 events of breast device infection/exposure were included in the analysis. Out of 26 cases of severe infection without prosthesis exposure, the implant was successfully salvaged in 8 (30.8%). On the other hand, none of the patients with severe infection and prosthesis exposure ( *n* = 7) had their device salvaged. Therefore, the literature supports the decision contained in our protocol to remove the implant of the patients with severe infection and prosthesis exposure.

The last parameter is if there is extensive tissue necrosis. In this situation, there is no possibility of approximating the viable tissues surrounding the lesion, and the implant needs to be removed. If there is no sign of severe infection, no history of radiotherapy, and no extensive tissue necrosis, a primary suture is indicated. This approach was successful in 8 out of 14 patients (57.1%) in the present study. Two studies 33 34 reported the use of a capsular flap to cover exposed implants after breast reconstruction. Brandstetter et al. 33 reported the case of a patient presenting with a small exposure of breast implant after a skin-sparing mastectomy. The patient was submitted to capsulotomy, removal of the implant, lavage of the pocket, and insertion of a new implant, which was covered with a capsular flap. 33 Varga et al. 34 reported 19 cases of patients submitted to capsuloplasty after implant exposure; they did not specified the extent of the exposure and, in most of cases, new implants were used. Our data demonstrates that performing a primary suture can prevent the patients from undergoing an invasive surgical procedure, and it is successful in most cases.

There are limitations to the present study. First, the small number of patients with implant exposure was insufficient to demonstrate which risk factors are associated with this complication. However, it is important to emphasize that this was not the aim of the present study, as other authors have already investigated this subject. Second, the protocol presented needs to be validated by other groups of surgeons, as well as the patients' acceptance of its proposals.

## Conclusion

Our clinical protocol combines the evidence from the literature, clinical and individualized data of the patient, and the experience of a surgeon specialized in breast reconstruction. This protocol, based on three key points (history of radiotherapy, severe infection, and extensive tissue necrosis), is a practical and potentially-reproducible method of managing one of the most common complications of implant-based breast reconstruction.
